# What Are Young Women Living Conditions after Breast Cancer? Health-Related Quality of Life, Sexual and Fertility Issues, Professional Reinsertion

**DOI:** 10.3390/cancers12061564

**Published:** 2020-06-12

**Authors:** Emerline L. F. Assogba, Ariane Mamguem Kamga, Helène Costaz, Clémentine Jankowski, Agnès Dumas, Patrick Roignot, Geneviève Jolimoy, Charles Coutant, Patrick Arveux, Tienhan Sandrine Dabakuyo-Yonli

**Affiliations:** 1 Breast and Gynaecologic Cancer Registry of Côte d’Or, Georges François Leclerc Comprehensive Cancer Centre, 1 rue Professeur Marion, 21000 Dijon, France; eassogba@cgfl.fr (E.L.F.A.); amamguem@cgfl.fr (A.M.K.); Patrick.Arveux@unisante.ch (P.A.); 2 Department of Surgical Oncology, Georges François Leclerc Comprehensive Cancer Centre, 1 rue Professeur Marion, 21000 Dijon, France; hcostaz@cgfl.fr (H.C.); cjankowski@cgfl.fr (C.J.); ccoutant@cgfl.fr (C.C.); 3 Clinical Epidemiology and Economic Evaluation Applied to Vulnerable Populations (Epidémiologie Clinique et Évaluation Économique appliquée aux Populations Vulnérables [ECEVE])—INSERM UMR 1123, University of Paris—Site Villemin, 10 avenue de Verdun, 75010 Paris, France; agnes.dumas@inserm.fr; 4 Pathology Centre, 33 rue Nicolas Bornier, 21000 Dijon, France; P.ROIGNOT@centre-de-pathologie.fr; 5 Burgundy Oncology Institute, 18 cours Général de Gaulle, 21000 Dijon, France; gjolimoy@yahoo.fr; 6 Faculty of Medecine and Pharmacy, Burgundy Franche-Comté University, 21000 Dijon, France; 7 Center for Primary Care and Public Health (Unisanté), University of Lausanne, 1010 Lausanne, Switzerland; 8 Lipids, Nutrition, Cancer Research Center, French National Institute of Health and Medical Research (Institut National de la Santé et de la Recherche Médicale [INSERM]) U1231, 21000 Dijon, France; 9 National Quality of Life and Cancer Clinical Research Platform, 21000 Dijon, France

**Keywords:** breast neoplasm, young women, health-related quality of life, fertility, sexuality, return to work

## Abstract

In recent decades, the living conditions of young breast cancer (BC) survivors have garnered increasing attention. This population-based study aimed to identify the clinical, social and economic determinants of Health-Related Quality of Life (HRQoL), and to describe other living conditions of young long-term BC survivors. Women with non-metastatic BC diagnosed between 2006 and 2015, aged 45 years and younger at the time of diagnosis, were identified through the Breast and Gynecologic Cancer Registry of the Côte d’Or, France. Participants completed self-report questionnaires including standardized measures of HRQoL, anxiety, depression, social deprivation, social support and sexuality. Fertility and professional reintegration issues were also assessed. The determinants of HRQoL were identified using mixed regression model. In total, 218 BC survivors participated in the survey. The main determinants of poor HRQoL were anxiety, depression, comorbidities, social deprivation and menopausal status. Among 72% of women who did not receive information about fertility preservation, 38% of them would have liked to have been informed. Finally, 39% of survivors reported a negative impact of BC on their professional activity. This study showed that BC stage or treatments did not have an impact on HRQOL of young long-term BC survivors. Fertility, sexuality and professional reintegration remained the main concerns for survivors. Specific interventions in these population should focus on these issues.

## 1. Introduction

Breast cancer (BC) is the most common cancer and the leading cause of death by cancer in women in France and around the world [1]. In France, the incidence of BC for women under 40 years of age is increasing with an average annual variation of +0.9 [2]. Concurrently, this increase in incidence is associated with a decrease in mortality, with an average annual variation of −1.6 [2]. Survival after BC varies from one country to another [3], with the highest survival rate (87%) being observed in France, from 2005 to 2010, compared to other European countries [4,5]. Improved survival raises the question of improved living conditions for survivors. In this regard, increasing attention has been focused on health-related quality of life (HRQoL) [6] in recent decades, as well as on issues related to fertility and socio-professional reintegration, particularly among young women. Indeed, cancer is affecting increasing numbers of working individuals, who have a long professional life ahead of them. A return to work is generally perceived by this population as important for recovery [7]. Therefore, it is necessary to focus on the professional reintegration of young women who have had a BC. Moreover, Meneses et al. reported that difficulties linked to maternity and fertility were the main factors hindering HRQoL in young BC survivors, due to the side effects of treatment, such as chemotherapy, on ovarian function [8]. The sexuality of women with BC can also be perturbed, for both physical and psychological reasons. To the best of our knowledge, few population-based studies have encompassed all these post-cancer issues among young women with BC in France.

Using data from the specialized Côte d’Or Breast and Gynaecologic Cancer Registry, this study aimed to identify the clinical, social and economic determinants of HRQoL among young BC survivors. Secondarily, we describe the living conditions of BC survivors, with regard to fertility, sexual function, psychological distress and professional reinsertion.

## 2. Materials and Methods

### 2.1. Patients

This cross-sectional study was conducted using data from the specialized Breast and Gynaecologic Cancer Registry of the Côte d’Or Department in France, the only registry in France to focus on breast and gynecological cancers. The registry catchment area has approximately 500,000 inhabitants, of whom 270,000 are women. The population is predominantly rural, with low migration. The registry has been collecting comprehensive population-based data at the time of diagnosis for all cases of breast and gynecological cancer occurring in Côte d’Or residents since 1982. The Côte-d’Or is one of the eight French departments in the region of Burgundy Franche-Comté, in northeast France. Its prefecture, Dijon, is also the regional capital. The department’s economic activity is 70% tertiary, 25% industry and 5% agriculture. Previous studies have been published using data from the French Côte d’Or Breast and Gynaecologic Cancer Registry [9,10]. Female BC survivors, aged 45 and younger at the time of diagnosis, diagnosed with non-metastatic BC between 1st January 2006 and 31st December 2015 were identified. Patients who died or relapsed before January 2019 were excluded. In February 2019, eligible participants were sent a study information pack by post that included an information letter, the study questionnaires and a stamped return envelope. Patient’s referring physicians were provided with information about the study and were informed that their patients would be approached for participation. For patients who did not respond within one month, a reminder was sent.

Ethics approval: this study was performed in accordance with the declaration of Helsinki. The study was approved by the French national data protection authority (CNIL-MR003 N°1989764-v0) and by the Committee for the Protection of Persons South-East I (2018-A03431-54).

### 2.2. Outcomes, Measures, Study Variables

#### 2.2.1. Outcome Variables

The main outcome of this study was HRQoL, as assessed by the validated Medical Outcome Study 12-item Short Form health survey questionnaire (SF-12) [11,12]. Through its 12 items, this questionnaire generates eight scales: physical functioning, role physical, bodily pain, role emotional, vitality, social functioning, mental health and general health. All scales were scored according to the standard scoring method of the SF-12 scoring manual. Each score ranges from 0 to 100, with higher scores representing a better level of HRQoL.

#### 2.2.2. Predictor Variables

The Hospital Anxiety and Depression Scale (HADS) questionnaire, validated and adapted in French by Lepine et al. [13] was used to detect anxiety and depressive disorders. This scale has 14 items (7 each for anxiety and depression), all rated from 0 to 3. Total scores range from 0 to 21, and a subscale score of 11 or more indicates presence of anxiety or depression.

Social support was assessed by Sarason’s social support questionnaire (SSQ6), validated and adapted in French by Rascle et al. [14]. This 6-item questionnaire measures the availability of social support and the satisfaction with the perceived support. Availability scores range from 0 to 54, and satisfaction scores range from 6 to 36. A higher satisfaction score represents better perceived social support. Scores were categorized into 2 classes, using the respective median value.

Socio-economic deprivation was assessed using the French Evaluation de le Précarité et des Inégalités de santé pour les Centres d’Examen de Santé (EPICES) [15]. This questionnaire, developed specially for the French context, contains 11 items that take into account the overall living conditions and generate an overall deprivation score. Scores vary from 0 to 100, and allow classification of patients as deprived or not deprived (>30 and ≤30, respectively).

The Female Sexual Function Index (FSFI) is a self-administrated questionnaire specific to sexual function in women. It was developed by Raymond Rosen and has been validated in French [16,17]. Through its 19 items, it explores six scales (desire, excitement, lubrication, orgasm, satisfaction and pain) of sexual function. Global score ranges from 2 to 36; an overall score <26.5 indicates sexual dysfunction. For each scale, a score <3.9 is considered as a deterioration on that scale.

Fertility concerns after BC were assessed using a specific questionnaire, developed in conjunction with clinicians and surgeons. Data collected included the number of pregnancies before and after diagnosis, information on treatment effects and fertility preservation before treatment, fertility preservation techniques, adoption, number of abortions and number of spontaneous miscarriages after treatment end.

Professional reinsertion was assessed using another study-specific questionnaire developed in conjunction with sociologists and psychologists. Data collected were problems obtaining loans, income since diagnosis, ability to work (after treatment and at the time of survey), impact of cancer and perceived discrimination in professional life.

Patient and tumor characteristics, including age at diagnosis (categorized as ≤35 and >35 years), age at the time of the survey (categorized as ≤45 and >45 years), Charlson’s comorbidity index, tumor stage, tumor grade, hormone status, human epidermal growth factor receptor 2 (HER2) status and treatments were extracted from the Côte d’Or Breast and Gynaecologic Cancer Registry database. Time since diagnosis was categorized in two classes according to the median (≤86 and >86 months). body mass index (BMI) was classified as underweight and normal weight (BMI ≤25 kg/m^2^) and overweight (BMI >25 kg/m^2^). Tumor stage was categorized according to the 8th edition of the Tumor Nodes Metastasis (TNM)-American Joint Commission of Cancer (AJCC) classification [18].

#### 2.2.3. Statistical Analysis

We compared clinical characteristics and treatments between respondents and non-respondents using the chi-square or Fischer’s exact test for categorical variables and the Mann-Whitney test for continuous variables. HRQoL, anxiety and depression, social support, deprivation and sexual function scores were generated, categorized and described, in addition to the other quantitative variables as mean (standard deviation, SD) or median (range). Clinical and social characteristics, treatment and all qualitative variables are described as number and percentage.

A mixed regression model was built to identify the determinants of the 8 domains of HRQoL. The variables eligible for multivariate analysis were those with a *p* value < 0.10 by univariate analysis (for the eight dimensions of SF-12). Correlations and interactions were tested for eligible variables. Results are reported as multivariate analysis coefficients, SDs and *p* values.

Bonferroni’s correction was used to adjust the α-risk in the eight multivariate models (α’ = α/n with n corresponding to the number of comparisons made). The significance level for the multivariate analyses was therefore set at *p* < 0.00625.

All analyses were performed using SAS software version 9.4 (SAS Institute Inc., Cary, NC, USA).

## 3. Results

**Figure 1:**
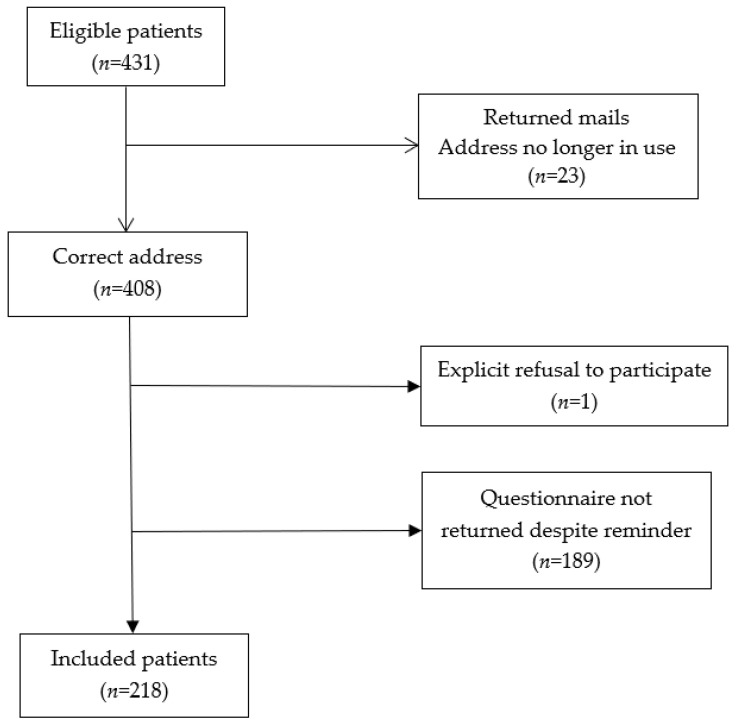
Study population flow-chart.

Four hundred and thirty-one patients with BC were eligible for this study. For 23 of these, the return address was no longer in use. The questionnaire was thus mailed to 408 participants, of whom 218 completed the questionnaire (response rate 53.4%). The details are shown in Figure 1.

Respondents and non-respondents did not differ significantly in terms of clinical characteristics or treatments: age at diagnosis (*p* = 0.0490); age at time of survey (*p* = 0.1015); time since diagnosis (*p* = 0.7607); AJCC stage (*p* = 0.2673); tumor grade (*p* = 0.3391); hormone receptor status (*p* = 0.1408); HER2 status (*p* = 0.5709); tumor triple negative status (*p* = 0.2479); menopausal status at diagnosis (*p* = 0.8060); Charlson comorbidity index (*p* = 0.2336); chemotherapy (*p* = 0.1477); radiotherapy (*p* = 0.4795); endocrine therapy (*p* = 0.5772). The details of comparisons between respondents and non-respondents are shown in Table S1.

### 3.1. Demographic and Clinical Characteristics of Participants

Mean age of study participants was 40.1 (SD = 4.4) years at the time of diagnosis and 47.4 (SD = 5.1) at the time of the survey. Mean BMI was 24.3 (SD = 4.5). Median time since diagnosis was 86 months (range, 36–155). At the time of diagnosis, 90.3% of women were married or living maritally. This proportion decreased to 60.9% at the time of survey. Other main characteristics were AJCC stage 1 (42%), no comorbidities (90%), menopaused at time of survey (53%), no deprivation (81%) and employed at the time of the survey (87%). All patients underwent surgery and the majority were treated by chemotherapy (77%), radiotherapy (85%) and endocrine therapy (71%). The demographic and clinical characteristics of the participants are shown in Table 1.

### 3.2. HRQoL, Sexual Function, Social Support, Anxiety and Depression Scores

#### 3.2.1. HRQoL Scores

The highest average score was in the physical functioning scale (80.8), and the lowest was in vitality (50.7). There were less than 3% of missing values in each of SF-12 dimensions (see Table 2).

#### 3.2.2. Sexual Function Scores

Mean global FSFI score was 22.1 (SD = 10.5). Using the threshold of 26.5 to define the presence of sexual dysfunction, approximately 55% of women reported sexual dysfunction. Moreover, a deterioration was seen for each subscale (desire, arousal, pain, lubrication, orgasm), except satisfaction (see Table 2).

#### 3.2.3. Social Support Scores

The median social support availability score was 19 (range, 0–54) and the median social support satisfaction score was 31 (range, 6–36) (see Table 2). A higher social support satisfaction score represents better perceived social support.

#### 3.2.4. Anxiety and Depression Scores

Using the threshold of 11 to define the presence of mood disorders, 29% of women had anxiety, and less than 10% had depression (see Table 2).

### 3.3. Determinants of HRQoL

Significant determinants of HRQoL in young women with BC are shown in Table 3. By multivariate analysis, depression was found to be a significant independent determinant of general health (β = 17.34; *p* = 0.0006), physical functioning (β = 20.34; *p* = 0.0028), role physical (β = 24.75; *p* = 0.0008), vitality (β = 29.22; *p* < 0.0001), role emotional (β = 32.62; *p* < 0.0001), mental health (β = 18.57; *p* < 0.0001) and social functioning (β = 26.67; *p* < 0.0001). Patients with anxiety were more likely to have bodily pain (β = 13.40; *p* = 0.0015), limitations due to emotional state (β = 20.03; *p* < 0.0001), poor mental health (β = 19.52; *p* < 0.0001) and poor social functioning (β = 15.90; *p* < 0.0001). Social support satisfaction was associated with general health (β = 7.30; *p* = 0.0042) and social functioning (β = 11.31; *p* = 0.0005). Women menopaused at time of survey were more likely to have deteriorated general health (β = 7.19; *p* = 0.0051). Deprivation was significantly associated with bodily pain (β = 13.44; *p* = 0.0037) and mental health (β = 9.40; *p* = 0.0013). Women with no comorbidities at the time of diagnosis were more likely to have better physical functioning (β = 6.03; *p* = 0.0055).

### 3.4. Fertility Data and Concerns

Before treatment, 65.1% of women had regular menstrual cycles. This rate decreased significantly after treatment to 17%. Forty-nine (22.8%) women had no menstrual cycles before treatment, and this increased to 121 (57.1%) after treatment. Seventy women (33%) reported that they did not receive information before treatment about the impact of cancer treatment on fertility and ovarian function, and 150 (72%) reported that they did not receive information about fertility preservation. Among these, 55 (38%) would have liked to have been informed.

Eighty-five percent of the study participants already had children at the time of diagnosis. Twenty-one (9.8%) had a pregnancy project at time of diagnosis and 66 women (33.2%) gave up a pregnancy project after the diagnosis. Three out of these 21 women had a medically assisted procreation (MAP) consultation before treatment, of whom two agreed with the proposed fertility preservation and one woman became pregnant with MAP after treatment. Two of these 21 women had a spontaneous pregnancy after treatment. The fertility data of the study population are shown in Table 4.

### 3.5. Professional Reinsertion

The full details of professional reinsertion are shown in Table 5. Sixty women (27%) declared that their income had decreased since diagnosis, and 154 (70%) patients had a bank loan in progress. Among 62 women who had sought a home loan since diagnosis, 47 (81%) reported difficulties (exclusion and/or increased insurance premium and refusal) with insurance. Eighty-two women (38%) reported a negative impact of cancer on their professional life and 160 women (73%) reported a decreased ability to work after treatment and at the time of the survey (59%). The main reason for the reduced ability to work was fatigue in 84% of women at the end of treatment, and 75% at the time of the survey. Thirty-seven percent of women reported the occurrence of one of the following events: retirement, stopped working, made redundant, bankruptcy, sale or cessation of an independent professional activity, resignation, different position and transfer within the same company; among whom 60% specified that the events occurred due to BC.

## 4. Discussion

This population-based study assessed the medical and socioeconomic determinants of HRQoL among young women with BC identified through the French Côte d’Or Breast and Gynaecologic Cancer Registry. One of the main strengths of this study was the use of a specialized registry database, which has the advantage of being representative of regionally treated patients, enabling us to assess long-term HRQoL. We also used validated instruments to assess HRQoL and psychological outcomes. Moreover, this study encompassed all aspects of post-cancer life in young women with BC (HRQoL, fertility, sexuality, professional reintegration).

The response rate (53.4%) in this survey was similar to that of previous population-based studies in this geographical area [19,20,21]. Indeed, Chu et al. and Dialla et al. reported a response rate of 59% and 62%, respectively, in studies including women of all ages at diagnosis of BC [20,21]. There was no significant difference between respondents and non-respondents in terms of clinical characteristics and treatments in our study, and therefore, no potential selection bias in our results.

The limitations of this study included the cross-sectional design. Indeed, the time since diagnosis ranged from 36 to 155 months in our study, with a median time since diagnosis of 86 months. This raises concerns about recall bias, given the questions about fertility and return to work events at diagnosis or during the time of treatment.

Our results showed that anxiety, depression, social support satisfaction, deprivation, comorbidities and menopause were significant determinants of HRQoL for long-term young BC survivors, as may also be the case in a population of young women without BC [22,23,24,25].

In this study, approximately half of the women had sexual dysfunction. Cobo-Cuenca et al. and Abril-Requena et al. also found similar results in a population of Spanish women with BC [26,27]. Sexual function is an important component of HRQoL among BC survivors. The effects of BC on sexuality depend on treatment, disease severity and how patients experienced their relationship and sexuality before cancer [28,29].

In our study, the participants reported disturbed menstrual cycles after treatment. This could be attributable to the side effects of BC treatment on ovarian function, such as adjuvant chemotherapy [8]. Jacobson et al. reported similar results in a population of women with BC treated by chemotherapy with or without radiotherapy in the United States [30]. We noted that among women who did not receive information about fertility preservation, more than a third would have liked to have been informed. This underlines the unmet need for fertility preservation information among this age group, as shown by our study. Young women need access to high-quality health information to support their involvement in medical decision-making concerning fertility preservation [31,32,33]. These results may be useful for clinical practice, in terms of counseling on fertility preservation as part of BC treatment. The very low rate of access to fertility preservation observed in our study should be interpreted in light of the period of diagnosis. Today, fertility preservation is accessible to larger numbers. Few women became pregnant after treatment in our study. Anderson et al. found that the chance of achieving a first pregnancy was lower for women >5 years after diagnosis of all cancer types, with a marked reduction in women with BC [34]. Nevertheless, we cannot rule out the possibility that the low number of pregnancies after treatment may also depend on the structure of the questionnaire used or some unexplored specificity of the population under study. Indeed, in our study, few women were interested in having children after diagnosis, and for that reason, the number of pregnancies and miscarriages were indeed very low.

Another important finding was that for half of the women in our study, their income was unchanged since the BC diagnosis. Saito et al. found a lower rate than in the present study [35]. One explanation could be that approximately 50% of our sample had a higher level of education. However, Jensen et al. found that, overall, after seven years, BC did not have any effect on income but the negative effect of BC on income lasted longer among women with a higher educational level [36]. In our study, about one-third of women with BC reported a negative impact of BC on their professional life. These results are consistent with previous studies on the influence of cancer on work-related issues among Japanese BC survivors aged 20 years and older and Korean BC survivors at working age [35,37]. The women in our study also reported a decreased ability to work after treatment, as well as difficulties obtaining loans. In women with BC, the main reason for the impaired ability to work was fatigue, both before (84%) and after treatment (75%). One explanation for this reduced ability to work due to fatigue in women with BC might be the treatment strategies [38]. Abrahams et al. found that cancer-related fatigue was reported in up to 90% of persons with cancer during adjuvant treatment [39]. Moreover, Jones et al. reported the persistence of fatigue up to 6 years post-treatment in approximately one-third of cancer survivors [40].

## 5. Conclusions

In conclusion, the results of this study show that the main determinants of HRQoL in long-term young BC survivors are anxiety, depression, social deprivation, social support satisfaction and menopausal status. Other main issues highlighted by this study are the negative impact of BC on professional reintegration and sexual function, the unmet need for information about the impact of cancer treatment on fertility, and for information about fertility preservation. Specific interventions in this population should therefore focus on the promotion of professional reintegration and information about the impact of cancer treatment on fertility, as well as fertility preservation.

## Figures and Tables

**Table 1:** Demographic and clinical characteristics of the study population (N = 218).

Characteristics	No. of Patients	Median (Min–Max)	%
Age at diagnosis, years			
≤35	27		12.4
>35	191		87.6
Age at time of survey, years			
≤45	60		27.5
>45	158		72.5
BMI at time of survey			
≤25	134		62.0
>25	82		38.0
Missing data	2		
Marital status at time of survey			
Married/living maritally	132		60.6
Single/divorced/widowed	86		39.4
Missing data	0		
Educational level			
Less than high school diploma	57		26.5
High school diploma or higher	158		73.5
Missing data	3		
Employment at time of survey			
Employed	189		87.5
Unemployed	27		12.5
Missing data	2		
Time since diagnosis, months			
Mean		87.7	
Median (min-max)	218	86 (36–155)	
SD		34.2	
Time since diagnosis, months			
≤86	110		50.5
>86	108		49.5
AJCC stage			
1	93		42.7
2/3	125		57.3
Missing data	0		
Tumor grade			
I	41		19.6
II	92		44.0
III	76		36.4
Missing data	9		
Hormone Receptor status			
Positive	166		76.5
Negative	51		23.5
Missing data	1		
HER2 status			
Positive	47		21.8
Negative	169		78.2
Missing data	2		
Triple negative status			
Yes	37		17.1
No	179		82.9
Missing data	2		
Menopausal status at time of survey			
Menopausal	112		53.6
Non-menopausal	97		46.4
Missing data	9		
Charlson comorbidity Index			
=0	195		90.3
≥1	21		9.7
Missing data	2		
Surgery			100
Yes	216		
No	0		
Missing data	2		
Chemotherapy			
Yes	168		77.4
No	49		22.6
Missing data	1		
Radiotherapy			
Yes	186		85.7
No	31		14.3
Missing data	1		
Endocrine therapy			
Yes	156		71.9
No	61		28.1
Missing data	1		
EPICES deprivation score ^a^	213		
Mean		17.3	
Median [min-max]		13.6 (0–75.7)	
SD		17.2	
Missing data	5		
EPICES deprivation score ^a^			
EPICES ≤ 30	174		81.7
EPICES > 30	39		18.3
Missing data	5		

^a^: Scores range from 0 to 100 and classify patients as deprived or not deprived (>30 and ≤30, respectively). BMI: body mass index. HER2: human epidermal growth factor receptor 2. AJCC: American Joint Commission of Cancer.

**Table 2:** Health-related quality of life (HRQoL), sexual function, social support, anxiety and depression scores in the study population (N = 218).

Dimensions	No. of Patients	Mean (SD)	Median (Min-Max)
**Health-Related Quality of Life: SF12 ^a^**			
General health	217	67.3 (19.1)	60 (25–100)
Physical functioning	217	80.8 (27.1)	100 (0–100)
Role physical	216	68.3 (28.2)	75 (0–100)
Role emotional	217	67.8 (26.6)	75 (0–100)
Bodily pain	217	75.0 (27.3)	75 (0–100)
Mental health	217	59.9 (19.6)	62.5 (0–100)
Vitality	217	50.7 (24.6)	50 (0–100)
Social functioning	215	68.5 (25.8)	75 (0–100)
**Sexual function ^b^**			
Desire	215	3 (1.3)	3 (1.2–6)
Arousal	213	3.2 (1.9)	3.6 (0–6)
Pain	211	3.6 (2.4)	4.4 (0–6)
Satisfaction	194	4.2 (1.7)	4.4 (0.8–6)
Lubrication	215	3.5 (2.3)	4.2 (0–6)
Orgasm	211	3.4 (2.2)	4 (0–6)
Global Score	188	22.1 (10.5)	25.1 (2–36)
Sexual dysfunction (%)			
Yes	104 (55.3)		
No	84 (44.7)		
Missing data	30		
**Social support ^c^**			
Social support availability	212	20.5 (11.2)	19 (0–54)
Social support satisfaction	196	28.8 (7.9)	31 (6–36)
Social support availability (%)			
<19	102 (48.1)		
≥19	110 (51.9)		
Missing data	6		
Social support satisfaction (%)			
<31	97 (49.5)		
≥31	99 (50.5)		
Missing data	22		
**HADS ^d^**			
Anxiety	216	8.7 (3.8)	8 (2–20)
Depression	217	4.7 (3.6)	4 (0–16)
Anxiety			
<11	153 (70.8)		
≥11	63 (29.1)		
Missing data	2		
Depression			
<11	198 (91.2)		
Dimensions	No. of Patients	Mean (SD)	Median (Min-Max)
≥11	19 (8.8)		
Missing data	1		

^a^: Scores range from 0 to 100 with higher scores representing better HRQoL. ^b^: Global score ranges from 2 to 36; an overall score <26.5 corresponds to sexual dysfunction. For each scale, a score <3.9 is considered as an alteration on that scale. ^c^: Availability scores range from 0 to 54 and satisfaction scores range from 6 to 36. A higher social support satisfaction score represents better perceived social support. ^d^: Both the anxiety and depression subscores range from 0 to 21, with a score of 11 or higher indicating the probable presence of mood disorder.

**Table 3:** Significant determinants of health-related quality of life.

Scales Scores of the SF-12 and Variables	Estimate	Standard Error	*p*-Value
**General health ^a^**			
Depression			0.0006
<11/≥11	17.34	4.99	
Social support satisfaction			0.0042
≥31/<31	7.30	2.52	
Menopausal status at time of survey			0.0051
No/Yes	7.19	2.53	
**Physical functioning ^b^**			
Depression			0.0028
<11/≥11	20.34	6.70	
Scales scores of the SF-12 and variables	Estimate	Standard error	*p*-value
Charlson’s comorbidity score			0.0055
=0/≥1	6.03	4.02	
**Role physical ^c^**			
Depression			0.0008
<11/≥11	24.75	7.23	
**Role emotional ^d^**			
Anxiety			<0.0001
<11/≥11	20.03	3.63	
Depression			<0.0001
<11/≥11	32.62	6.26	
**Bodily pain ^e^**			
Anxiety			0.0015
<11/≥11	13.40	4.15	
EPICES deprivation score			0.0037
≤30/>30	13.44	6.96	
**Mental health ^f^**			
Anxiety			<0.0001
<11/≥11	19.52	2.58	
Depression			<0.0001
<11/≥11	18.57	4.04	
EPICES deprivation score			0.0013
≤30/>30	9.40	2.87	
**Vitality ^g^**			
Depression			<0.0001
<11/≥11	29.22	5.82	
**Social functioning ^h^**			
Anxiety			<0.0001
<11/≥11	15.90	3.74	
Depression			<0.0001
<11/≥11	26.67	6.25	
Social support satisfaction			0.0005
≥31/<31	11.31	3.21	

Mixed models regression (Significant at *p*-value < 0.00625) ^a^: Adjusted for anxiety, Evaluation de le Précarité et des Inégalités de santé pour les Centres d’Examen de Santé (EPICES) deprivation score, social support availability, Charlson’s comorbidity score at diagnosis. ^b^: Adjusted for age at the time of diagnosis, time since diagnosis, body mass index (BMI), anxiety, EPICES deprivation score, social support availability, tumor stage, hormonal status, menopausal status at the time of the survey. ^c^: Adjusted for anxiety, EPICES deprivation score, social support availability, social support satisfaction, Charlson’s comorbidity score at diagnosis, menopausal status at time of survey. ^d^: Adjusted for time since diagnosis, EPICES deprivation score, social support satisfaction, radiotherapy, menopausal status at the time of the survey. ^e^: Adjusted for time since diagnosis, depression, social support availability, social support satisfaction, marital status at the time of diagnosis, tumor stage. ^f^: Adjusted for time since diagnosis, social support availability. ^g^: Adjusted for time since diagnosis, anxiety, EPICES deprivation score, social support availability, menopausal status at the time of the survey. ^h^: Adjusted for time since diagnosis, EPICES deprivation score, social support availability.

**Table 4:** Fertility data of the study population.

Variables	No. of Patients	%
Contraception at time of diagnosis		
No	44	20.9
Yes	167	79.2
Missing data	7	
Menstrual cycle before treatment		
Non-existent	49	22.8
Regular	140	65.1
Irregular	26	12.1
Missing data	3	
Menstrual cycle after treatment		
Non-existent	121	57.1
Regular	36	17.0
Irregular	55	25.9
Missing data	6	
Children at the time of diagnosis		
No	32	14.8
Yes	184	85.2
Missing data	2	
Pregnancy project at time of diagnosis		
No	194	90.2
Yes	21	9.8
Missing data	3	
Gave up a pregnancy project after treatment		
Yes	66	33.2
No	133	66.8
Missing data	19	
Planned pregnancy project after treatment		
Yes	15	7.3
No	190	92.7
Missing data	13	
Children at time of diagnosis among women who had pregnancy project at time of diagnosis (*n* = 21)		
No	7	33.3
Yes	14	66.7
Spontaneous pregnancy before diagnosis (*n* = 14)		
No	1	7.1
Yes	13	92.9
Pregnancy with MAP before diagnosis (*n* = 14)		
0	7	77.8
1	2	22.2
Missing data	5	
Information received about impact of treatment on fertility and ovarian function		
Yes	70	33.8
No	68	32.9
I forgot	69	33.3
Missing data	11	
Fertility preservation-related information		
Yes	56	27.2
No	150	72.8
Missing data	12	
Would have liked to be informed if fertility preservation-related information not given (*n* = 150)		
Yes	55	38.7
No	87	61.3
Missing data	8	
MAP Consultation (*n* = 21)		
No	16	84.2
Yes	3	15.8
Missing data	2	
Fertility preservation proposed if MAP consultation ^a^ (*n* = 3)		
No	0	
Yes	3	100
Agreed to proposed fertility preservation (*n* = 3)		
No	1	66.7
Yes	2	33.3
Spontaneous pregnancy after treatment (*n* = 21)		
0	13	86.7
1	2	13.3
Missing data	6	
Pregnancy with MAP after treatment (*n* = 21)		
0	13	92.9
1	1	7.1
Missing data	7	
Adoption (*n* = 21)		
Yes	1	5.3
No	18	94.7
Missing data	2	
Abortion since cancer (*n* = 21)		
Yes	0	
No	20	100
Missing data	1	
Spontaneous miscarriages (*n* = 21)		
Yes	1	5.0
No	19	95.0
Missing data	1	

^a^: MAP: Medically Assisted Procreation.

**Table 5:** Professional outcomes in the study population.

Variables	No. of Patients	%
Income since cancer diagnosis		
Increased	43	19.8
Unchanged	114	52.5
Decreased	60	27.7
Missing data	1	
Bank loan in progress		
No	64	29.4
Yes	154	70.6
Difficulties repaying bank loans in progress		
No	139	64.9
Yes	28	13.1
Not concerned	47	22.0
Missing data	4	
Asked for a loan since treatment		
No	94	45.2
Yes	114	54.8
Missing data	10	
Answer to a home loan request (*n* = 61) ^a^		
Agreement without conditions	10	17.5
Difficulties with insurance (refusal, exclusions and/or increased premium)	47	82.5
Missing data	4	
Employment at time of diagnosis		
Employed	183	84.3
Unemployed	34	15.7
Missing data	1	
Arduous working conditions at time of diagnosis		
Yes	91	46.2
No	106	53.8
Missing data	21	
Decreased ability to work after the end of treatment		
Yes	160	73.4
No	53	24.3
Not concerned	5	2.3
Reasons for decreased ability to work after the end of treatment		
Fatigue	135	84.4
Pain	69	43.1
Limitation of some of my movements	69	43.1
Limitation of my cognitive abilities	80	50.0
Emotional problems	58	36.3
Other symptoms	10	6.3
Decreased ability to work at the time of survey		
Yes	128	59.0
No	85	39.2
Not concerned	4	1.8
Missing data	1	
Reasons for decreased ability to work at the time of the survey		
Fatigue	96	75.0
Pain	53	41.4
Limitation of some of my movements	51	39.8
Limitation of my cognitive abilities	54	42.2
Emotional problems	39	30.5
Other symptoms	4	3.1
Impact of cancer on work		
Positive	45	21.2
Negative	82	38.7
None	85	40.1
Missing	6	
Perceived discrimination		
Yes	61	28.4
No	123	57.2
Not applicable	31	14.4
Missing data	3	
Events since the end of treatment		
Retirement, stopped working	1	0.5
Made redundant	22	10.8
Bankruptcy, sale or cessation of an independent activity	6	3.0
Resignation	11	5.4
Different position, transfer (within the same company)	36	17.7
None	127	62.6
Missing data	15	
Role of cancer or its after-effects in the aforementioned events (*n* = 76) ^b^		
No	30	39.5
Yes	46	60.5
Missing data		

^a^: patients who had had estate loan ^b^: patients who had had at least one of the aforementioned events.

## Supplementary Materials

The following are available online at https://www.mdpi.com/2072-6694/12/6/1564/s1, Table S1: Comparison of clinical characteristics between respondents and non-respondents.

## References

[1] Bray F., Ferlay J., Soerjomataram I., Siegel R.L., Torre L.A., Jemal A. (2018). Global cancer statistics 2018: GLOBOCAN estimates of incidence and mortality worldwide for 36 cancers in 185 countries. CA Cancer J. Clin..

[2] Defossez G., Le Guyader-Peyrou S., Uhry Z., Grosclaude P., Colonna M., Dantony E., Remontet L., Monnereau A., Woronof A.-S., Delafosse P. (2019). Estimations Nationales de L’incidence et de la Mortalité par Cancer en France Métropolitaine Entre 1990 et 2018. Étude à Partir des Registres des Cancers du Réseau Francim. Résultats Préliminaires.

[3] Coleman M.P., Quaresma M., Berrino F., Lutz J.M., De Angelis R., Capocaccia R., John L., Koifman S., Storm H.H., Sant M. (2008). Cancer survival in five continents: A worldwide population-based study (CONCORD). Lancet Oncol..

[4] Cowppli-Bony A., Uhry Z., Remontet L., Guizard A.V., Voirin N., Monnereau A., Bouvier A.M., Colonna M., Bossard N., Woronoff A.S. (2016). Survie des Patients Atteints de Cancer en France, 1989-2013.–Etude à Partir des Registres des Cancers du Réseau Francim Partie 1–Tumeurs Solides.

[5] De Angelis R., Sant M., Coleman M.P., Francisci S., Baili P., Pierannunzio D., Trama A., Nennecke A., Siseling S., Berrino F. (2014). Cancer survival in Europe 1999–2007 by country and age: Results of EUROCARE-5—A population-based study. Lancet Oncol..

[6] Baumann C., Briançon S., Metz V. (2010). Maladie chronique et qualité de vie: Enjeux, définition et mesure. Actualité Dossier Santé Publique.

[7] Duijts S.F., Kieffer J.M., van Muijen P., van der Beek A. (2017). Sustained employability and health-related quality of life in cancer survivors up to four years after diagnosis. Acta Oncol..

[8] Meneses K., McNees P., Azuero A., Jukkala A. (2010). Evaluation of the Fertility and Cancer Project (FCP) among young breast cancer survivors. Psychooncology.

[9] Auguste A., Cortet M., Dabakuyo-Yonli T.S., Launay L., Arnould L., Desmoulins I., Roignot P., Arveoux P., Bertaut A., Poillot M.-L. (2017). Breast cancer subtype of French women is not influencedby socioeconomic status: A population based-study. PLoS ONE.

[10] Dialla P.O., Quipourt V., Gentil J., Marilier S., Poillot M.L., Roignot P., Dabakuyo-Yonli T.S., Arveux P., Guiu S., Darut-Jouve A. (2015). In breast cancer, are treatments and survival the same whatever a patient’s age? A population-based study over the period 1998–2009. Geriatr. Gerontol. Int..

[11] Gandek B., Ware J.E., Aaronson N.K., Apolone G., Bjorner J.B., Brazier J.E., Sullivan M., Prieto L., Leplege A., Kaasa S. (1998). Cross-validation of item selection and scoring for the SF-12 Health Survey in nine countries: Results from the IQOLA Project. International Quality of Life Assessment. J. Clin. Epidemiol..

[12] Ware J.E., Kosinski M., Turner-Bowker D.M., Gandek B. (2005). How to Score Version 2 of the SF-12 Health Survey: With a Supplement Documenting Version 1.

[13] Lepine J.P., Godchau M., Brun P. (1985). Anxiety and depression in patients. Lancet.

[14] Rascle N., Bruchon-Schweitzer M., Sarason I.G. (2005). Short Form of Sarason’s Social Support Questionnaire: French Adaptation and Validation. Psychol. Rep..

[15] Sass C., Moulin J.J., Guéguen R., Abric L., Dauphinot V., Dupré C., La Rosa E., Magnier P., Lebbe E., Guenot C. (2006). Le score Epices: Un score individuel de précarité. Construction du score et mesure des relations avec des données de santé, dans une population de 197389 personnes. Bull. Epidemiol. Hebdomadaire.

[16] Rosen R., Brown J., Heiman S., Leiblum C., Meston R., Shabsigh D., Ferguson R., D’Agostino R. (2000). The Female Sexual Function Index (FSFI): A multidimensional self-report instrument for the assessment of female sexual function. J Sex Marital Ther.

[17] Wylomanski S., Bouquin R., Philippe H.J., Poulin Y., Hanf M., Dréno B., Quéreux G., Rouzier R. (2014). Psychometric properties of the French Female Sexual Function Index (FSFI). Qual. Life Res..

[18] Amin M.B., Greene F.L., Edge S.B., Compton C.C., Gershenwald J.E., Brookland R.K., Winchester D.P., Byrd D.R., Mayer L., Gress D.M. (2017). The eighth edition AJCC cancer staging manual: Continuing to build a bridge from a population-based to a more “personalized” approach to cancer staging. CA Cancer J. Clin..

[19] Mamguem Kamga A., Dumas A., Joly F., Billa O., Simon J., Poillot M.L., Dabakuyo-Yonli T.S., Jouve A.D., Fumoleau P., Countant C. (2019). Long-Term Gynecological Cancer Survivors in Côte d’Or: Health-Related Quality of Life and Living Conditions. Oncologist.

[20] Chu W.O., Dialla P.O., Roignot P., Bone-Lepinoy M.C., Poillot M.L., Coutant C., Dabakuyo-Yonli T.S., Arveux P. (2016). Determinants of quality of life among long-term breast cancer survivors. Qual. Life Res..

[21] Dialla P.O., Chu W.O., Roignot P., Bone-Lepinoy M.C., Poillot M.L., Coutant C., Dabakuyo-Yonli T.S., Arveux P. (2015). Impact of age-related socio-economic and clinical determinants of quality of life among long-term breast cancer survivors. Maturitas.

[22] Short H., Al Sayah F., Ohinmaa A., Lahtinen M., Johnson J.A. (2018). The relationship of neighbourhood-level material and social deprivation with health-related quality of life. Qual Life Res..

[23] Johansson R., Carlbring P., Heedman A., Paxling B., Andersson G. (2013). Depression, anxiety and their comorbidity in the Swedish general population: Point prevalence and the effect on health-related quality of life. PeerJ.

[24] Helgeson V.S. (2003). Social support and quality of life. Qual. Life Res..

[25] Schultz P.N., Klein M.J., Beck M.L., Stava C., Sellin R.V. (2005). Breast cancer: Relationship between menopausal symptoms, physiologic health effects of cancer treatment and physical constraints on quality of life in long-term survivors. J. Clin. Nurs..

[26] Cobo-Cuenca A.I., Martin-Espinosa N.M., Sampietro-Crespo A., Rodríguez-Borrego M.A., Carmona-Torres J.M. (2018). Sexual dysfunction in Spanish women with breast cancer. PLoS ONE.

[27] Abril-Requena A., García-Torres F., Alós F.J. (2019). Sexual dysfunction and phobic anxiety in breast cancer survivors. Psycho Oncology.

[28] Dizon D.S. (2009). Quality of Life after Breast Cancer: Survivorship and Sexuality. Breast J..

[29] Raggio G.A., Butryn M.L., Arigo D., Mikorski R., Palmer S.C. (2014). Prevalence and correlates of sexual morbidity in long-term breast cancer survivors. Psychol. Health.

[30] Jacobson M.H., Mertens A.C., Spencer J.B., Manatunga A.K., Howards P.P. (2016). Menses resumption after cancer treatment-induced amenorrhea occurs early or not at all. Fertil. Steril..

[31] Chin H.B., Howards P.P., Kramer M.R., Mertens A.C., Spencer J.B. (2016). Which Female Cancer Patients Fail to Receive Fertility Counseling Before Treatment in the State of Georgia. Fertil. Steril..

[32] Jukkala A.M., Azuero A., McNees P., Bates G.W., Meneses K. (2010). Self-assessed knowledge of treatment and fertility preservation in young women with breast cancer. Fertil. Steril..

[33] Thewes B., Meiser B., Taylor A., Phillips K.A., Pendlebury S., Capp A., Dalley D., Goldstein D., Baber R., Friedlander M.L. (2005). Fertility-and menopause-related information needs of younger women with a diagnosis of early breast cancer. J. Clin. Oncol..

[34] Anderson R.A., Brewster D.H., Wood R., Nowell S., Fischbacher C., Kelsey T.W., Wallace W.H.B. (2018). The impact of cancer on subsequent chance of pregnancy: A population-based analysis. Hum. Reprod..

[35] Saito N., Takahashi M., Sairenchi T., Muto T. (2014). The impact of breast cancer on employment among Japanese women. J. Occup. Health.

[36] Jensen L.S., Overgaard C., Bøggild H., Garne J.P., Lund T., Overvad K., Fonager K. (2017). The long-term financial consequences of breast cancer: A Danish registry-based cohort study. BMC Public Health.

[37] Ahn E., Cho J., Shin D.W., Park B.W., Ahn S.H., Noh D.Y., Yun Y.H., Lee E.S., Nam S.J. (2009). Impact of breast cancer diagnosis and treatment on work-related life and factors affecting them. Breast Cancer Res. Treat..

[38] Schmidt M.E., Scherer S., Wiskemann J., Steindorf K. (2019). Return to work after breast cancer: The role of treatment-related side effects and potential impact on quality of life. Eur. J. Cancer. Care..

[39] Abrahams H.J., Gielissen M.F., Schmits I.C., Verhagen C.A., Rovers M.M., Knoop H. (2016). Risk factors, prevalence, and course of severe fatigue after breast cancer treatment: A meta-analysis involving 12 327 breast cancer survivors. Ann. Oncol..

[40] Jones J.M., Olson K., Catton P., Catton C.N., Fleshner N.E., Krzyzanowska M.K., McCready D.R., Wong R.K.S., Jiang H., Howel D. (2016). Cancer-related fatigue and associated disability in post-treatment cancer survivors. J. Cancer Surviv..

