# Supplementary material for: What Are Young Women Living Conditions after Breast Cancer? Health-Related Quality of Life, Sexual and Fertility Issues, Professional Reinsertion
Source: Cancers (Basel). 2020 Jun 12;12(6):1564. doi: 10.3390/cancers12061564 (PMC7353050; doi:10.3390/cancers12061564)
Supplement: Supplementary file 1 [file cancers-12-01564-s001.pdf]

Supplementary Materials

What Are Young Women Living Conditions after Breast Cancer? Health-Related Quality of Life, Sexual and Fertility Issues, Professional Reinsertion

Emerline L. F. Assogba, Ariane Mamguem Kamga, Helène Costaz, Clémentine Jankowski, Agnès Dumas, Patrick Roignot, Geneviève Jolimoy, Charles Coutant, Patrick Arveux and Tienhan Sandrine Dabakuyo-Yonli

Table S1. Comparison of clinical characteristics between respondents and non-respondents.

Characteristics	Respondents		Non-Respondents		*p*-Value
	n = 218	%	*n* = 190	%	
Age at diagnosis, years					0.0490[a]
Mean (SD)	40.1 (4.4)		40.9(3.9)		
Median [min–max]	41 [22–45]		42 [28–45]		
Missing data	0		0		
Age at time of survey, years					0.1015[a]
Mean (SD)	47.4 (5.1)		48.5 (4.8)		
Median [min - max]	48 [28–57]		49 [32–58]		
Missing data	0		0		
Time since diagnosis, months					0.4296[a]
Mean (SD)	87.7 (34.2)		90.2 (33.1)		
Median [min - max]	86 [36–155]		88.5 [37–155]		
Missing data	0		0		
Time since diagnosis, months					0.7607[b]
≤ 86	110	50.5	93	48.9	
> 86	108	49.5	97	51.1	
Missing data	0		0		
AJCC stage					0.2673[b]
1	93	42.7	91	48.2	
2/3	125	57.3	98	51.8	
Missing data	0		1		
Tumor grade					0.3991[b]
I	41	19.6	33	17.7	
II	92	44.0	95	50.8	
III	76	36.4	59	31.5	
Missing data	9		3		
RH status					0.1408[b]
Positive	166	76.5	155	82.4	
Negative	51	23.5	33	17.6	
Missing data	1		2		
HER2 status					0.5709[b]
Positive	47	21.8	36	19.5	
Negative	169	78.2	149	80.5	

Characteristics	Respondents		Non-Respondents		*p*-Value
	$n = 218$	%	$n = 190$	%	
Missing data	2		5		
Tumor triple status					0.2479[b]
Yes	37	17.1	24	13.0	
No	179	82.9	161	87.0	
Missing data	2		5		
Menopausal status at diagnosis					0.8060[b]
Menopausal	4	2.0	4	2.3	
Non-menopausal	199	98.0	167	97.7	
Missing data	15		19		
Charlson comorbidity Index					0.2336[b]
= 0	195	90.4	174	93.6	
≥ 1	21	9.6	12	6.4	
Missing data	2		4		
Surgery					
Yes	216	100	188	100	
No	0	0	0	0	
Missing data	2		2		
Chemotherapy					0.1477b
Yes	168	77.4	133	71.1	
No	49	22.6	54	28.9	
Missing data	1		3		
Radiotherapy					0.4795b
Yes	186	85.7	163	88.1	
No	31	14.3	22	11.9	
Missing data	1		5		
Endocrine therapy					0.5772b
Yes	156	71.9	129	69.4	
No	61	28.1	57	30.6	
Missing data	1		4		

Significant at $p < 0.05$, a: Mann-Whitney test, b: chi-square test, HER2: Human Epidermal Growth Factor Receptor 2, AJCC: American Joint Commission of Cancer.

© 2020 by the authors. Licensee MDPI, Basel, Switzerland. This article is an open access article distributed under the terms and conditions of the Creative Commons Attribution (CC BY) license (http://creativecommons.org/licenses/by/4.0/).
